# Patients Are Using Dietary Supplement for the Treatment of Their Diseases without Consultation with Their Physicians and Pharmacists

**DOI:** 10.3390/pharmacy11060179

**Published:** 2023-11-17

**Authors:** Tsuyoshi Chiba

**Affiliations:** National Institute of Health and Nutrition, National Institutes of Biomedical Innovation, Health and Nutrition, Osaka 566-0002, Japan; tyschiba@nibiohn.go.jp; Tel.: +81-6-6384-1120

**Keywords:** consultation, dietary supplements, diseases, patients, treatment

## Abstract

Inappropriate use of dietary supplements by patients might exacerbate their diseases. The aim of this study was to clarify the situation of dietary supplement use for disease treatment among patients. A cross-sectional online questionnaire survey was conducted between 18 and 30 November 2022. A preliminary survey revealed that the proportion of patients who used dietary supplements to treat their disease ranged from 7.7% (cancer) to 28.2% (menopausal disorder). In the actual survey, across all diseases, patients who used dietary supplements for treatment purposes were satisfied with their therapeutic effects. Information obtained via the internet was the most common reason given for starting to use supplements. The proportions of patients who used supplements concurrently with medicine ranged from 25.4% (liver disease) to 72.2% (mental disorder). Most users of supplements did not consult with their physicians or pharmacists about them. However, patients preferred face-to-face consultation with a physician or pharmacist when discussing the concomitant use of medicines. In conclusion, the role of pharmacists is important for the appropriate use of dietary supplements among patients, especially concomitant use with medicines. More awareness about dietary supplements is needed for healthcare professionals to consult with their patients.

## 1. Introduction

Appropriate use of dietary supplements is encouraged as a self-care procedure. However, it has been reported that patients use dietary supplements, including various herbal products, for the treatment of their diseases [1,2]. The use of dietary supplements has been reported among patients diagnosed with eye disease, high plasma cholesterol levels, arthritis, and cancer [3]. It appears that this situation may be the result of a flood of internet misinformation about health—including claims of therapeutic effects of dietary supplements [4,5,6].

The use of dietary supplements for therapeutic purposes may result not only in missed opportunities for appropriate treatments but also in the exacerbation of disease. Moreover, in the case of patients taking pharmaceuticals, interactions between dietary supplements and medicines may occur during digestion, absorption, distribution, and metabolism, with consequent risks to health. Cytochromes P450 (CYP) are a major source of variability in drug-metabolized enzymes and mainly exist in the liver and other tissues. In humans, CYP1, 2, and 3 families are responsible for the drug metabolism in clinical use. The most expressed forms are CYP3A4, CYP2C9, CYP2C8, and CYP2E1 in the liver [7]. In this regard, CYPs are an important target for interactions between dietary supplements and medicines. For example, it is well known that vitamin K interferes with the anti-coagulant effects of warfarin [8]. However, vitamin K is not the only food component that interacts with warfarin, which is mainly metabolized by CYP1A2, CYP2C8, CYP2C9, CYP2C19, and CYP3A4 in the liver; food components that affect these CYPs also affect the efficacy of warfarin [8]. As with warfarin, there have been many reports of adverse interactions with other medicines induced by the consumption of various foods and herbs as supplements [9,10,11]. In addition, it has been reported that certain herbs can themselves cause liver dysfunction [12]. In light of these findings, attention must be paid to the use of dietary supplements by patients.

However, it is also known that dietary supplements, especially vitamins, minerals, and amino acids, are helpful to support nutritional conditions in elderly people [13] and cancer patients [14,15]. Such individuals cannot obtain enough nutrients from a regular diet because of decreased appetite, polypharmacy, dementia, frailty, poor dentition, or swallowing difficulties [16]. In addition, prescribed medications may interfere with micronutrient absorption and induce nutritional insufficiencies [17]. When treating and curing individuals affected by disease, it is important that good nutritional conditions are maintained [18,19]. However, even vitamins, minerals, and amino acids can be harmful when taken excessively or when they interact negatively with medicines. For this reason, consultations with healthcare professionals, such as physicians or pharmacists, are important when determining the appropriate use of dietary supplements. However, researchers have found that most patients use dietary supplements without consulting their physicians or pharmacists [1,2,20,21] and that physicians and pharmacists do not always ask their patients about their use of dietary supplements [20,22]. Communication between healthcare professionals and patients is therefore important for the prevention of adverse events caused by inappropriate use of dietary supplements.

In this study, an internet survey was conducted to learn more about the motives, attitudes, and beliefs of patients regarding their usage of dietary supplements for disease treatment and how they perceived their situation regarding consultation with health professionals about their supplement usage.

## 2. Materials and Methods

### 2.1. Surveillance

A cross-sectional online questionnaire survey was conducted on Japanese residents by Cross Marketing Inc. (Tokyo, Japan) between 18 and 30 November 2022. An invitation e-mail for survey cooperation with a webpage link to the survey form was sent to computer-randomized survey monitors aged 20 to 79 years who belonged to the survey monitor panel of Cross Marketing Inc. The questionnaire consisted of a preliminary survey and an actual survey. An explanation of the study was provided on the first page of the preliminary survey form, and only those individuals who agreed to participate answered the questionnaire. The preliminary survey was conducted on 44,605 respondents to select eligible persons who used dietary supplements for the purpose of treating their disease, and the responses of 1500 respondents were collected for the actual survey. This study was conducted with the approval of the Research Ethics Committee of the National Institutes of Biomedical Innovation, Health and Nutrition (No. 382-2, approved on 9 August 2022) and in accordance with the Declaration of Helsinki.

### 2.2. Questionnaire

The preliminary survey contained questions about medical history, time of diagnosis, use of dietary supplements for therapeutic purposes, and motives for supplement usage. The actual survey contained questions about the realization of the therapeutic effect of dietary supplements, perceptions of dietary supplements, the situation of consultation with physicians and pharmacists, the concurrent use of medications, and preferred methods of consultation about the concomitant use of dietary supplements and medicines (File S1: Questionnaire).

### 2.3. Statistical Analyses

Differences in distribution among groups were compared using the chi-squared (χ^2^) test. A *p*-value of <0.05 was considered statistically significant. After conducting the chi-squared test, a residual analysis was conducted to determine which categories had significant proportional differences. All statistical analyses were performed using IBM SPSS Statistics ver.28.0.1.0 (IBM Corporation, Armonk, NY, USA).

## 3. Results

### 3.1. Preliminary Survey

A total of 44,605 respondents, including 22,822 males and 21,783 females aged 20–79 years, were recruited to identify those who used dietary supplements to treat their diseases (Table 1). The results showed that the prevalence of dietary supplement use for therapeutic purposes varied across diseases, being highest for menopause (28.2%), followed by liver disease (14.6%), kidney disease (14.5%), and rheumatoid arthritis (13.3%), with cancer (7.7%) ranking lowest. Across all diseases, the prevalence of dietary supplement use for therapeutic purposes was higher in patients diagnosed within the previous six months, compared with patients diagnosed more than six months previously (data not shown).

### 3.2. Characteristics of the Respondents in the Actual Survey

The actual survey was conducted on 1500 eligible participants from the preliminary survey who were categorized by sex (814 males and 686 females) and age (66 in their 20 s, 171 in their 30 s, 333 in their 40 s, 458 in their 50 s, 405 in their 60 s, and 167 in their 70 s). If a single patient was diagnosed with multiple diseases and used dietary supplements for each disease, that patient was redundantly included in each disease for which patients used dietary supplements (Table 1).

### 3.3. Motives for Beginning to Use Dietary Supplements for Therapeutic Purposes

Motives for beginning to use dietary supplements for therapeutic purposes are shown in Table 2. The internet (product sites, review sites, etc.) was the most common source of information (40.9%), followed by television or radio (25.3%), package claims for products (15.5%), and others. Recommendations from healthcare professionals (physicians, pharmacists, or dieticians) (14.5%) and from clerks at pharmacies or drugstores (7.3%) were also reported. The contributions made by the social networking services of Twitter, Instagram, and Facebook were low, at 3.8%, 3.3%, and 3.8%, respectively, high amongst those in younger generations (20 s and 30 s), and low in older generations (60–70 s). In addition, healthcare professionals were also high in younger generations (20 s and 40 s) and lower in older generations (60 s).

### 3.4. Realization of the Therapeutic Effect of Dietary Supplements

The realization of the therapeutic effect of dietary supplements on each disease is shown in Table 3. When the responses “It is very helpful” and “It is partially helpful” were combined, with the exception of those affected by kidney disease, more than half of patients felt that dietary supplements were effective for their disease, with totals ranging from 57.1% for liver disease to 75.1% for rheumatoid arthritis. Even among kidney disease patients, the proportion was slightly less than half, at 48.4%. However, small percentages of patients, ranging from 1.4% of dyslipidemia sufferers to 9.8% of those suffering from coronary heart disease, answered “It is not helpful at all”, even though they were using dietary supplements. Although significant differences were observed in the distribution of realization levels by disease, no trends were observed depending on the characteristics of the disease (e.g., health checkup items and recognizable symptoms, such as pain).

### 3.5. Perception of Dietary Supplements

The perception of dietary supplements in patients who use them for therapeutic purposes is shown in Table 4. More than half of respondents thought that dietary supplements were safe and involved fewer adverse events compared with medicines. They also thought that dietary supplements were effective for the prevention of diseases. In addition, 47.0% of patients agreed and 13.1% strongly agreed that “Dietary supplements can be used concomitantly with medicines”. Only a small proportion of patients were concerned about the concomitant use of dietary supplements and medicines, with 8.2% disagreeing and 3.5% strongly disagreeing with the statement above. However, only 27.4% of patients agreed and 4.9% strongly agreed that “Dietary supplements can treat disease”. Finally, more than half of patients distinguished between dietary supplements and medicines.

### 3.6. Consultation with Physicians or Pharmacists

One-third of patients consulted their physicians (38.6%) or their pharmacists (29.7%), as shown in Table 5. The main reasons given for not consulting were almost the same for both physicians and pharmacists, and they included the following: “I do not have to tell, because it is just food.”, “I have never been asked.”, and “I have been using it without any problems”. In addition, some patients did not seek consultation because “Physicians/pharmacies will deny it and tell me to stop using it”. or “Physicians/pharmacies will get angry”.

### 3.7. Concomitant Use of Dietary Supplements and Medicines

The medication status of patients who used dietary supplements for the relevant disease is shown in Table 6. Among patients who used dietary supplements for therapeutic purposes, the prevalence of medications varied across their diseases. The highest prevalence was found for mental disorders (72.2%), followed by hypertension (67.6%), diabetes mellitus (65.1%), and coronary heart disease (61.0%). The lowest prevalence was for liver disease (25.4%).

### 3.8. Attitude Regarding the Continuation of Dietary Supplement Use

After providing information about the risk of interaction between dietary supplements and medicines to patients as follows: “Since dietary supplements are not medicines, they do not have therapeutic effects. Also, if you take medicines at the same time, the efficacy of the medicines may be strengthened or weakened, which may interfere with your medication.”, patients were asked whether they would continue with their dietary supplement usage. About half of patients answered, “Keep using”, and only 5.5% of them answered, “Stop using it immediately” (Table 7). There was a significant difference between users and non-users of medicines, so that the response “Decide after consultation with physicians/pharmacists” was higher and “Stop using a dietary supplement after running out of it” was lower among medicine users compared with non-users. However, the response “Keep using” was given by almost the same proportions of medicine users (51.5%) and non-users (49.2%).

### 3.9. Consultation about the Concomitant Use of Dietary Supplements and Medicines

Table 8 shows different kinds of healthcare professionals and the methods of consultation about concomitant use of dietary supplements and medicines that are preferred by patients. When asked about professionals, most patients answered physicians (64.2%) or pharmacists (54.3%), though a small number gave the response “I check up by myself (e.g., the internet and books)” (13.6%). Concerning preferred method of consultation, a majority of patients answered “Face-to-face” (73.5%), and demand for other consultation methods was relatively low.

## 4. Discussion

This study surveyed awareness of dietary supplement use and consultation status among patients who used dietary supplements for the therapeutic treatment of their disease. The prevalence of dietary supplement use varied across diseases, ranging from 7.7% in cancer to 28.2% in menopausal disorder and from 57.1% in liver disease to 75.1% in rheumatoid arthritis, according to patients who were satisfied with the efficacy of dietary supplements. Furthermore, one-third of patients concomitantly used medicines to treat the diseases that they also used dietary supplements for, and they wished to continue using dietary supplements even after they were provided with risk information about the interaction between dietary supplements and medicines. Despite this, most of them preferred face-to-face consultations with physicians or pharmacists.

The widespread use of complementary and alternative medicines (CAMs) by cancer patients, mainly in the form of herbal medicines and dietary supplements, has been previously reported [14,23,24,25,26]. However, in the present study, only 7.7% of cancer patients used dietary supplements. One of the reasons for this discrepancy might be the small number of cancer patients in this survey in the first place, and it may be that these individuals were not serious cancer patients because they participated in an internet questionnaire survey. It has been reported that CAM utilization tends to be higher among patients in advanced cancer stages [27]. Therefore, it is conceivable that the utilization rate of dietary supplements was unusually low in this study. Second, Agaricus blazei mushroom had been the most popular ingredient for CAM in Japan, but the use of Agaricus blazei supplements has induced severe hepatic dysfunction in cancer patients [28], and the Ministry of Health, Labour and Welfare has issued a warning to this effect. Because this was also picked up by the media, this might have affected cancer patients’ perceptions of dietary supplements. However, details of the types and stages of their cancers were not asked. Further research is needed to clarify the actual use of dietary supplements by cancer patients, considering their types and stages.

The highest prevalence of dietary supplement use was among menopausal disorder patients, with a total of 28.2%. In a previous survey on the use of supplements claiming female-hormone-like effects, treatment for menopausal symptoms was the most common purpose for which these products were used [29]. Phytoestrogens (e.g., soy isoflavones, daidzein, red clover isoflavones, and flaxseed extract) are plant-derived compounds that have a similar structure to estradiol and affinity to the estrogen receptor [30]. Phytoestrogen-containing supplements are therefore sold with the claim that they have female hormone-like effects and thus improve symptoms of menopausal disorder. In addition, Japanese people are familiar with soy products, such as tofu, miso, and natto [31,32]. It is thought that this circumstance may lower barriers to the use of soy supplements for menopausal disorders.

The internet was the most common source of information for patients seeking to begin the use of dietary supplements for the therapeutic treatment of their diseases. It has been reported that cancer patients gained information about CAM via the internet and SNS (Facebook) [26]. At present, to claim therapeutic effects on food labeling and advertisements is illegal in Japan; nevertheless, many such claims for supplements are made on websites without scientific evidence. It is conceivable that people believe such claims and purchase dietary supplements as a result, even though these products actually have no curative effect. In addition, it has been reported that more than half of the health-related information provided by healthcare professionals on Twitter was false [33]. In addition, the media provides commercials and advertisements for dietary supplements that healthcare professionals recommend, and some of them might be misleading. This situation might be a reason that healthcare professionals could be the motive for dietary supplement use for therapeutic purposes in this study. Misinformation on the website was highlighted during the COVID-19 pandemic and was termed an “infodemic” by the World Health Organization [34]. Fake news related to COVID-19 prevention (e.g., consumption of salty water, bleach, or garlic for prevention of coronavirus infection) was compounded by low levels of awareness, knowledge, and health literacy; in this regard, SNS (especially Twitter) was found to be the major platform of dissemination [35].

One of the important findings in this study is that more than half of patients felt that dietary supplements produced therapeutic effects against their diseases, even when the supplements had no such effects. The placebo effect seems to be the main reason why patients reported therapeutic effects of dietary supplements [36,37]. They also believed that dietary supplements were safe. Such perceptions might have encouraged them to continue with the use of dietary supplements and medicines, even after they were provided with risk information about the interaction between dietary supplements and medicines. Despite this, patients in this survey still wished to consult with physicians and pharmacists on a face-to-face basis. More consultation with healthcare professionals could improve this situation. However, knowledge about dietary supplements amongst such professionals is inadequate, and healthcare professionals lack confidence in consulting about dietary supplements [38,39]. Indeed, 14.5% of respondents in our survey began to use dietary supplements for therapeutic purposes after receiving recommendations from healthcare professionals. Even though some dietary supplements, especially vitamin/mineral supplements, might be helpful for nutritional treatments in patients, the use of dietary supplements for therapeutic purposes is not recommended. Better education about dietary supplements is needed for healthcare professionals.

There are some limitations to this study. First, patients’ status, whether they were inpatients or outpatients, was not asked. This might affect the prevalence and purpose of dietary supplement use [1]. However, the majority of participants in our actual survey were patients with dyslipidemia, hypertension, mental disorders, and diabetes mellitus, so it is supposed that most patients were outpatients. Second, there was no question about the clinical efficacy of dietary supplement use. Even though patients reported satisfaction with the efficacy of their dietary supplements, most of them did not declare their supplement usage to their physicians or pharmacists, and it was unclear whether dietary supplements were actually effective for therapeutic purposes. Communication between healthcare professionals and patients is important for the appropriate use of dietary supplements. Lastly, because this survey was conducted via the internet, caution should be exercised in generalizing the results, especially with respect to older generations.

## 5. Conclusions

This study showed that the actual use of dietary supplements for therapeutic purposes was 57.1% (liver disease)–75.1% (rheumatoid arthritis), according to patients who were satisfied with the efficacy of the supplements that they used. This study also revealed that patients obtained the information via the internet and used dietary supplements to treat their diseases without consultation with their physicians or pharmacists. It is necessary to create an environment where patients can easily consult with physicians and pharmacists about dietary supplements, and physicians and pharmacists should acquire knowledge about dietary supplements and skills for consultation.

## Figures and Tables

**Table 1:** Prevalence of dietary supplement use among patients with different diseases.

			DS Users		Actual Survey
	*n* ^1^	Mean Age	*n*	%	*n* ^2^
Hypertension	9598	59.3	995	10.4	407
Diabetes mellitus	3872	58.1	461	11.9	209
Dyslipidemia	6433	57.4	840	13.1	420
Liver disease	1201	52.1	175	14.6	63
Kidney disease	543	52.2	79	14.5	31
Cerebrovascular disease	680	54.7	82	12.1	27
Coronary heart disease	1295	58.8	115	8.9	41
Rheumatoid arthritis	503	54.9	67	13.3	32
Mental disorder	4329	45.9	552	12.8	216
Menopausal disorder	794	51.0	224	28.2	131
Cancers	1415	56.2	109	7.7	50
Others	4736	52.6	434	9.2	230

DS; dietary supplement. Multiple answers. ^1^ *n* = 44,605 in the preliminary survey. ^2^ *n* = 1500 in the actual survey, who used dietary supplements for the purpose of treating their disease from the preliminary survey.

**Table 2:** Motives for using dietary supplements for therapeutic purposes by patient age groups.

	Total	20s	30s	40s	50s	60s	70s	*p*-Value
Internet (product sites, review sites, etc.)	40.9	30.3 ^#^	30.4 ^#^	42.0	43.2 *	44.9 *	39.5	0.014
Television or radio	25.3	21.2	22.8	21.9	27.5	27.9	25.1	0.368
Package claims for products	15.5	18.2	16.4	19.8	13.5	15.4	10.8	0.095
Healthcare professionals	14.5	28.8 *	15.2	18.9 *	14.2	7.2 ^#^	13.8	<0.001
Family, friends, and acquaintances	14.4	18.2	23.4 *	12.6	12.7	11.5	17.4	0.004
Newspapers, magazines, or advertisements	12.9	13.6	8.2 ^#^	9.0 ^#^	12.0	14.4	24.6 *	<0.001
POPs at the stores	7.9	9.1	13.5 *	9.3	6.8	5.9	5.4	0.032
Clerks at the pharmacy or drugstore	7.3	19.7 *	11.1	8.1	6.8	3.0 ^#^	6.6	<0.001
Twitter	3.8	19.7 *	7.6 *	5.1	2.2 ^#^	1.3 ^#^	0.0 ^#^	<0.001
Instagram	3.3	7.6 *	9.9 *	5.4 *	1.7 ^#^	0.7 ^#^	0.0 ^#^	<0.001
Facebook	2.1	7.6 *	5.3 *	3.3	1.3	0.3 ^#^	0.0 ^#^	<0.001
Others	5.0	3.0	3.5	6.0	4.4	6.6	4.2	0.518

POP; point-of-purchase. Multiple answers. The results are shown as percentages (%). Differences among ages were examined using the chi-squared test. After conducting the chi-squared test, a residual analysis was conducted to determine which categories had significant proportional differences. * Significantly more, ^#^ significantly less.

**Table 3:** Realization of the therapeutic effect of dietary supplements.

	*n*	It Is Very Helpful	It Is Partially Helpful	I Do Not Know	It May Not Be Helpful	It Is Not Helpful at All	*p* Value
Hypertension	407	12.5	48.4	32.9	4.2	2.0	0.019
Diabetes mellitus	209	17.7	45.0	30.6	4.3	2.4	
Dyslipidemia	420	13.6	46.7	35.7	2.6	1.4	
Liver disease	63	11.1	46.0	34.9	6.3	1.6	
Kidney disease	31	25.8	22.6	35.5	9.7	6.5	
Cerebrovascular disease	27	18.5	40.7	33.3	0.0	7.4	
Coronary heart disease	41	24.4	41.5	22.0	2.4	9.8	
Rheumatoid arthritis	32	31.3	43.8	15.6	6.3	3.1	
Mental disorder	216	16.7	40.7	33.8	5.1	3.7	
Menopausal disorder	131	15.3	53.4	26.0	3.8	1.5	
Cancers	50	20.0	42.0	32.0	2.0	4.0	
Others	230	13.9	43.9	37.8	3.9	0.4	

The results are shown as percentages (%). Differences in distribution among diseases were examined using the chi-squared test.

**Table 4:** Perception of dietary supplements.

	Strongly Agree	Agree	Neither Agree nor Disagree	Disagree	Strongly Disagree
DSs are safe because they are just food items.	9.4	50.4	28.5	8.2	3.5
DSs made from natural ingredients or herbs are safe.	8.9	42.0	36.6	9.1	3.3
DSs cause fewer adverse events than medicines.	16.7	45.9	26.5	7.9	2.9
DSs can be expected to be effective.	6.0	40.5	38.6	12.0	2.9
DSs can be used concomitantly with medicines.	13.1	47.0	28.2	8.2	3.5
DSs can prevent diseases.	7.3	43.5	35.7	10.1	3.3
DSs can treat diseases.	4.9	27.4	45.7	16.7	5.3
DSs can compensate for an unbalanced diet.	7.2	36.3	38.9	13.2	4.3
It may be hard to take every day.	5.7	22.9	27.0	28.9	15.4
DSs are indistinguishable from medicines.	2.8	12.7	32.0	30.6	21.9
DSs are expensive.	19.1	35.1	27.1	13.1	5.5

DSs; dietary supplements. The results are shown as percentages (%).

**Table 5:** Consultation with physicians or pharmacists.

	With Physicians	With Pharmacists
Yes, I do.	38.6	29.7
No, I do not.	61.4	70.3
Reasons for not consulting:		
I do not have to tell, because it is just food.	44.6	40.5
I have never been asked.	40.8	41.7
I have been using it without any problems.	29.0	23.9
It is hard to tell about dietary supplement use.	11.0	8.1
I use it only occasionally.	5.9	6.2
Physicians/pharmacists will deny it and tell me to stop using it.	5.6	2.0
I told dietary supplement use to pharmacists/physicians.	3.0	8.2
Physicians/pharmacists will get angry.	1.6	0.7
Others.	3.1	3.5

The results are shown as percentages (%). Multiple answers in “Reasons for not consulting”.

**Table 6:** Concomitant use of dietary supplements and medicines.

	DS Only *n* ^1^	DS + Medicine *n* ^2^	% ^3^
Hypertension	407	275	67.6
Diabetes mellitus	209	136	65.1
Dyslipidemia	420	254	60.5
Liver disease	63	16	25.4
Kidney disease	31	9	29.0
Cerebrovascular disease	27	15	55.6
Coronary heart disease	41	25	61.0
Rheumatoid arthritis	32	10	31.3
Mental disorder	216	156	72.2
Menopausal disorder	131	44	33.6
Cancers	50	14	28.0
Others	230	168	73.0

DSs; dietary supplements. ^1^ Patients who use dietary supplements for the relevant disease (*n* = 1500). ^2^ Patients who use dietary supplements and medicines for the relevant disease (*n* = 1122). ^3^ Percentage of DS + medicine (n^2^) to DS only (n^1^).

**Table 7:** Attitude regarding continuation of dietary supplement use after provision of information about the risk of interaction between dietary supplements and medicines.

	Total	Medicine Users ^1^	Medicine Non-Users ^2^	*p*-Value
Keep using	50.9	51.5	49.2	<0.001
Decide after consultation with physicians/pharmacists	29.5	31.4	24.1	
Stop using a dietary supplement after running out of it	12.2	9.6	19.8	
Stop using it immediately	5.5	4.9	6.9	
Others	1.9	2.6	0.0	

^1^ Patients who use dietary supplements and medicines for the relevant disease (*n* = 1122). ^2^ Patients who use only dietary supplements for the relevant disease (*n* = 378). The results are shown as percentages (%). Differences in distribution between medicine users and non-users were examined using the chi-squared test.

**Table 8:** Preferred methods of consultation about concomitant use of dietary supplements and medicines.

	*n*	%
Professionals		
Physicians	963	64.2
Pharmacists	814	54.3
Distributors of dietary supplements	160	10.7
Advisory staff ^1^	113	7.5
Registered dietitians	67	4.5
Staff at public health centers	33	2.2
Others	13	0.9
I check up by myself (e.g., the internet and books)	204	13.6
Styles		
Face-to-face	1103	73.5
E-mail	317	21.1
Web	259	17.3
Telephone	250	16.7
Application program (e.g., Line)	208	13.9
At public health centers	64	4.3
Others	30	2.0

Multiple answers. ^1^ Advisory staff is a unique Japanese license for a dietary supplement advisor who provides accurate information so that consumers can safely and appropriately select/use dietary supplements according to their dietary situation and health condition.

## Data Availability

The data presented in this study are available upon request from the corresponding author.

## Supplementary Materials

The following supporting information can be downloaded at: https://www.mdpi.com/article/10.3390/pharmacy11060179/s1. File S1: Questionnaire.
