# Supplementary material for: Patients Are Using Dietary Supplement for the Treatment of Their Diseases without Consultation with Their Physicians and Pharmacists
Source: Pharmacy (Basel). 2023 Nov 17;11(6):179. doi: 10.3390/pharmacy11060179 (PMC10661250; doi:10.3390/pharmacy11060179)
Supplement: Supplementary file 1 [file pharmacy-11-00179-s001.zip › pharmacy-2708046-SI.pdf]

Questionnaire

<u>Preliminary survey</u>

SC1. Have you been diagnosed with following diseases?

1. No, I have never diagnosed with diseases.
2. Hypertension
3. Diabetes mellitus
4. Dyslipidemia (high cholesterol and triglycerides)
5. Liver disease (hepatitis, fatty liver, liver dysfunction)
6. Kidney disease (renal failure, nephrosis, etc.)
7. Cerebrovascular disease (infarction, hemorrhage, subarachnoid hemorrhage, etc.)
8. Coronary heart disease (myocardial infarction, angina pectoris, arteriosclerosis, etc.)
9. Rheumatoid arthritis
10. Mental disorder (depression, insomnia, etc.)
11. Menopause disorder
12. Cancers
13. Others

SC2. When have you been diagnosed?

(Displayed only diseases that participant chose at SC1)

	Within a month	1 to 6 months ago	6 months to 1 year ago	1 to 3 years ago	More than 3 years ago
Hypertension					
Diabetes mellitus					
Dyslipidemia					
Liver disease					
Kidney disease					
Cerebrovascular disease					
Coronary heart disease					
Rheumatoid arthritis					
Mental disorder					
Menopause disorder					
Cancers					
Others					

SC3. Are you using dietary supplement to your diseases?

(Displayed only diseases that participant chose at SC1)

	Yes	No
Hypertension		
Diabetes mellitus		
Dyslipidemia		
Liver disease		
Kidney disease		
Cerebrovascular disease		
Coronary heart disease		
Rheumatoid arthritis		
Mental disorder		
Menopause disorder		
Cancers		
Others		

Actual survey

Q1. What was motive for using dietary supplements for therapeutic purpose?

1. Television or radio
2. Newspaper, magazines, or advertisements
3. Internet (product sites, review sites, word-of-mouth sites, etc.)
4. Facebook
4. Twitter
4. Instagram
5. POPs at the stores
6. package claims of products
7. Recommendations from family, friends, or acquaintances
8. Recommendations from clerks at pharmacies or drugstores
9. Recommendations from healthcare professionals (doctors, pharmacists, dieticians, etc.)
10. Others

Q2. Do you think that dietary supplements are helpful to treat your disease?

1. It is very helpful.
2. It is partially helpful.
3. I do not know.
4. It may not be helpful.
5. It is not helpful at all.

Q3 How do you think about dietary supplement?

	Strongly agree	agree	Neither agree nor disagree	Disagree	Strongly disagree
Dietary supplements are safe because they are just food items.					
Dietary supplements made from natural ingredients or herbs are safe.					
Dietary supplements cause less adverse events than medicines.					
Dietary supplements can be expected to be effective.					
Dietary supplements can be used concomitantly with medicines.					
Dietary supplements can prevent diseases.					
Dietary supplements can treat diseases.					
Dietary supplements can compensate for an unbalanced diet.					
It may be hard to take every day.					
Dietary supplements are indistinguishable from medicines.					
Dietary supplements are expensive.					

Q4. Do you tell your doctor about dietary supplement use?

If not, what is the reason?

1. Yes, I do.
2. No, I do not.
    - 2-1. I do not have to tell, because it is just food.
    - 2-2. I have been using it without any problems.
    - 2-3. I use it only occasionally.
    - 2-4. I have never been asked.
    - 2-5. It is hard to tell about dietary supplement use.
    - 2-6. Doctors will deny it, and tell me to stop using.
    - 2-7. Doctors will get angry.
    - 2-8. I told dietary supplement use to pharmacist.
    - 2-9. Others

Q5. Do you tell your pharmacist about dietary supplement use?

If not, what is the reason?

1. Yes, I do.
2. No, I do not.
   - 2-1. I do not have to tell, because it is just food.
   - 2-2. I have been using it without any problems.
   - 2-3. I use it only occasionally.
   - 2-4. I have never been asked.
   - 2-5. It is hard to tell about dietary supplement use.
   - 2-6. Doctors will deny it, and tell me to stop using.
   - 2-7. Doctors will get angry.
   - 2-8. I told dietary supplement use to doctor.
   - 2-9. Others

Q6. Are you taking medicines to treat your diseases?

(Displayed only diseases that participant chose at SC1)

	Yes	No
Hypertension		
Diabetes mellitus		
Dyslipidemia		
Liver disease		
Kidney disease		
Cerebrovascular disease		
Coronary heart disease		
Rheumatoid arthritis		
Mental disorder		
Menopause disorder		
Cancers		
Others		

Q7. "Since dietary supplements are not medicines, they do not have therapeutic effects. Also, if you take medicines at the same time, the efficacy of the medicines may be strengthened or weakened, which may interfere with your medication."
Will you keep dietary supplement use?

1. Keep using.
2. Decide after consultation with doctors/pharmacists
3. Stop using dietary supplement after running out it
4. Stop using immediately
5. Others

Q8. Who would you prefer to talk about concomitant use of dietary supplements and medicines?

1. Doctors
2. Pharmacists
3. Registered dietitians
4. Advisory staff
5. Distributors of dietary supplements
6. Staff at public health centers
7. I check up by myself (e.g., internet, books)
8. Others

Q9. Which is the best way to consult about concomitant use of dietary supplements and medicines?

1. Face-to-face
2. Web
3. Telephone
4. E-mail
5. Application program (e.g., Line)
6. At public health centers
7. Others

Thank you for your cooperation
